# Supplementary material for: Active site specificity profiling datasets of matrix metalloproteinases (MMPs) 1, 2, 3, 7, 8, 9, 12, 13 and 14
Source: Data Brief. 2016 Feb 22;7:299–310. doi: 10.1016/j.dib.2016.02.036 (PMC4777984; doi:10.1016/j.dib.2016.02.036)
Supplement: Supplementary file 10 — Supplementary material [file mmc10.zip › WebPICS_hMMP8_G_1%/subsite_coop.html]

 

PICS results


- # About
- # Manual
- # Analysis
- # Results

- Redraw
- Seqlogo
- Dependency
- Coop

**Potential subsite cooperativity analysis for hMMP8\_G\_1%**   
Minimum difference for subsite dependency was set to +- 10 percentage-points  
Subsite dependency was checked for all positional occurences above 1 x natural abundance  
**Subsite dependency check is restricted to P3 - P3'**

|  |  |  |  |
| --- | --- | --- | --- |
| Fixed residue | Affected residue(s) | Change (percentage-points) | Vice-Versa change |
| P3\_A | P1\_A | 14.2 | 26.2 |
| P3\_H | P1\_P | 52.5 | 29.1 |
| P3\_H | P1prime\_F | 54.2 | 38.7 |
| P3\_H | P2\_W | 56.7 | 70.8 |
| P3\_H | P2prime\_Q | 50.0 | 20.8 |
| P3\_H | P3prime\_V | 51.7 | 25.8 |
| P3\_I | P2\_L | 31.1 | 17.5 |
| P3\_I | P2prime\_H | 18.0 | 32.5 |
| P3\_I | P2prime\_R | 15.5 | 17.5 |
| P3\_I | P3prime\_A | 45.0 | 15.6 |
| P3\_P | P1\_N | -15.6 | -19.0 |
| P3\_P | P1prime\_L | -37.2 | -21.3 |
| P3\_P | P1prime\_Q | 18.6 | 43.4 |
| P3\_P | P1prime\_V | 15.0 | 35.0 |
| P3\_P | P2\_K | 12.2 | 31.2 |
| P3\_V | P2\_A | 11.4 | 10.6 |
| P3\_V | P2\_N | 10.4 | 22.5 |
| P3\_V | P2prime\_Q | 13.1 | 14.2 |
| P2\_A | P1\_A | 10.6 | 11.4 |
| P2\_A | P1\_Q | 12.2 | 15.6 |
| P2\_A | P1prime\_Q | 18.6 | 21.6 |
| P2\_A | P1prime\_Y | 11.0 | 38.3 |
| P2\_A | P3prime\_S | 11.4 | 13.3 |
| P2\_F | P1\_Q | 35.2 | 28.9 |
| P2\_F | P1prime\_F | 16.4 | 21.1 |
| P2\_F | P1prime\_V | 23.3 | 17.5 |
| P2\_F | P2prime\_I | 22.5 | 15.6 |
| P2\_F | P2prime\_V | 20.0 | 11.3 |
| P2\_F | P3prime\_N | 25.0 | 22.5 |
| P2\_H | P1prime\_Y | 30.0 | 22.5 |
| P2\_H | P3prime\_Q | 27.5 | 11.8 |
| P2\_K | P1\_A | 34.7 | 29.3 |
| P2\_K | P2prime\_K | 33.7 | 14.8 |
| P2\_L | P1\_N | 18.3 | 12.8 |
| P2\_L | P1\_P | 11.3 | 20.0 |
| P2\_L | P1prime\_C | 13.0 | 29.6 |
| P2\_M | P1prime\_V | 40.0 | 13.4 |
| P2\_Q | P3prime\_Q | 19.2 | 32.9 |
| P2\_W | P1\_P | 67.5 | 30.0 |
| P2\_W | P1prime\_F | 69.2 | 39.6 |
| P2\_W | P2prime\_Q | 90.0 | 30.0 |
| P2\_W | P3prime\_V | 66.7 | 26.7 |
| P2\_Y | P1prime\_C | 19.2 | 21.9 |
| P1\_A | P1prime\_Q | 20.8 | 22.5 |
| P1\_A | P2prime\_K | 33.0 | 17.2 |
| P1\_A | P3prime\_G | 29.6 | 13.3 |
| P1\_N | P1prime\_F | 11.6 | 37.9 |
| P1\_N | P2prime\_K | -12.1 | -11.2 |
| P1\_P | P1prime\_F | 27.5 | 35.4 |
| P1\_P | P2prime\_Q | 34.4 | 25.8 |
| P1\_P | P3prime\_V | 25.0 | 22.5 |
| P1\_Q | P1prime\_V | 17.3 | 15.8 |
| P1\_S | P2prime\_V | 15.3 | 13.3 |
| P1prime\_C | P3prime\_V | 20.3 | 14.2 |
| P1prime\_F | P2prime\_Q | 32.9 | 19.2 |
| P1prime\_F | P3prime\_V | 34.6 | 24.2 |
| P1prime\_L | P2prime\_K | 11.9 | 23.2 |
| P1prime\_V | P2prime\_I | 14.2 | 13.1 |
| P1prime\_V | P3prime\_N | 16.7 | 20.0 |
| P2prime\_I | P3prime\_N | 22.5 | 29.2 |
| P2prime\_K | P3prime\_G | 19.8 | 17.1 |
| P2prime\_Q | P3prime\_V | 25.0 | 30.0 |

  
**PICS analysis of protease: hMMP8\_G\_1%**  
120 cleavage sites analyzed  
PICS library made with (T)rypsin, (G)luC or (C)hymotrypsin:   
Cutoff for graphic display: 2 x natural abundance  

|  |  |
| --- | --- |
| Positional occurences  (table for total and relative (in %) values) | Occurences relative to natural abundance (table) |
|  |  |
